# Isolation, Screening, and Identification of Cellulolytic Bacteria from Natural Reserves in the Subtropical Region of China and Optimization of Cellulase Production by *Paenibacillus terrae* ME27-1

**DOI:** 10.1155/2014/512497

**Published:** 2014-06-19

**Authors:** Yan-Ling Liang, Zheng Zhang, Min Wu, Yuan Wu, Jia-Xun Feng

**Affiliations:** State Key Laboratory for Conservation and Utilization of Subtropical Agro-Bioresources, Key Laboratory of Ministry of Education for Microbial and Plant Genetic Engineering, College of Life Science and Technology, Guangxi University, 100 Daxue Road, Nanning, Guangxi 530004, China

## Abstract

From different natural reserves in the subtropical region of China, a total of 245 aerobic bacterial strains were isolated on agar plates containing sugarcane bagasse pulp as the sole carbon source. Of the 245 strains, 22 showed hydrolyzing zones on agar plates containing carboxymethyl cellulose after Congo-red staining. Molecular identification showed that the 22 strains belonged to 10 different genera, with the *Burkholderia* genus exhibiting the highest strain diversity and accounting for 36.36% of all the 22 strains. Three isolates among the 22 strains showed higher carboxymethyl cellulase (CMCase) activity, and isolate ME27-1 exhibited the highest CMCase activity in liquid culture. The strain ME27-1 was identified as *Paenibacillus terrae* on the basis of 16S rRNA gene sequence analysis as well as physiological and biochemical properties. The optimum pH and temperature for CMCase activity produced by the strain ME27-1 were 5.5 and 50°C, respectively, and the enzyme was stable at a wide pH range of 5.0–9.5. A 12-fold improvement in the CMCase activity (2.08 U/mL) of ME27-1 was obtained under optimal conditions for CMCase production. Thus, this study provided further information about the diversity of cellulose-degrading bacteria in the subtropical region of China and found *P. terrae* ME27-1 to be highly cellulolytic.

## 1. Introduction

With decades of studies on cellulose bioconversion, cellulases have been playing an important role in producing fermentable sugars from lignocellulosic biomass. Usually, cellulases are mainly composed of three types of synergistic enzymes: endoglucanases (EC 3.2.1.4) that hydrolyze the exposed cellulose chains of the cellulose polymer, exoglucanases (cellobiohydrolases, EC 3.2.1.91) that act to release cellobiose from the reducing and nonreducing ends, and *β*-glucosidases (EC 3.2.1.21) that help to cleave the cellobiose and short-chain cello-oligosaccharide into glucose [1].

Numerous microorganisms that are able to degrade cellulose have been isolated and identified. However, many studies have put more emphasis on fungi because the cellulases that they produce are abundant and easy to extract, and some of the fungal cellulases have been used as commercial cellulase [2]. Although fungi such as* Trichoderma*,* Aspergillus*,* Penicillium*,* Phanerochaete,* and* Fomitopsis* have been widely studied in recent years, researchers have also been paying attention to various bacteria that produce cellulases because of their fast growth, expression of multienzyme complexes, and resistance to extreme environments [3–8]. Bacteria belonging to the genera* Clostridium*,* Cellulomonas*,* Cellulosimicrobium*,* Thermomonospora*,* Bacillus*,* Ruminococcus*,* Erwinia*,* Bacteriodes*,* Acetovibrio*,* Streptomyces*,* Microbispora*,* Fibrobacter*, and* Paenibacillus* have been observed to produce different kinds of cellulase when incubated under anaerobic or aerobic conditions [4, 9, 10].

Several studies have been carried out to investigate the carboxymethyl cellulase (CMCase) activity of aerobic bacteria. For instance, a maximum CMCase activity (0.48 U/mL) of* Acinetobacter anitratus* was observed in the late logarithm phase [11]. Rastogi et al. reported that a maximum CMCase activity of 0.02 and 0.058 U/mL was exhibited by* Brevibacillus* sp. DUSELG12 and* Geobacillus* sp. DUSELR7 on days 10 and 7, respectively [12]. Furthermore, Gupta et al. isolated several cellulose-degrading bacteria exhibiting CMCase activities in the range of 0.162–0.400 U/mL [13].

With regard to studies on optimization of cellulase production by aerobic bacteria, Deka et al. used response surface methodology and found that* Bacillus subtilis* AS3 exhibited a maximum CMCase activity of 0.43 U/mL [14]. Furthermore, using response surface methodology and orthogonal experiment design for medium optimization, Da Vinha et al. and Sheng et al. observed a maximum CMCase activity of 2.0 and 1.432 U/mL by* Streptomyces viridobrunneus* SCPE-09 and* Pseudomonas* sp. HP207, respectively [15, 16]. Thus, isolation of aerobic bacterial strains producing higher cellulase activity is gaining increasing interest.

In this study, diverse aerobic bacteria capable of hydrolyzing cellulose were isolated from the subtropical region of China, with* Burkholderia* sp. being the most ubiquitous. Furthermore, a bacterial strain ME27-1, producing CMCase at 2.08 U/mL after optimization of culture conditions, was isolated and identified.

## 2. Materials and Methods

### 2.1. Collection of Soil Samples

The soil samples used in this study were collected from Maoer Mountain (Guilin City), Longgang (Chongzuo City), Dawang Ridge (Baise City), Huaping (Guilin City), Shankou Halodrymium (Beihai City) Natural Reserves, a starch factory in Fangchenggang City, a bagasse compost at the experimental farm of Guangxi University (Nanning City) in Guangxi Zhuang Autonomous Region, China, and Baima Snow Mountain Natural Reserve in Yunnan Province, China. The samples were taken from organic-rich soil.

### 2.2. Strain Isolation and Screening

The soil sample suspensions were inoculated on Czapek's medium [17] containing sugarcane bagasse pulp (in g/L: NaNO_3_, 2; MgSO_4_ *·*7H_2_O, 0.5; NaCl, 0.5; FeSO_4_ *·*7H_2_O, 0.01; KH_2_PO_4_, 1.0; yeast extract, 0.4; pulp, 5 (containing 80% water); and agar, 15.0; pH 5.0) and incubated at 28°C. Subsequently, single colonies were picked using an inoculating needle and inoculated onto Mandels and Reese medium [18] containing carboxymethyl cellulose sodium salt (CMC-Na; in g/L: KH_2_PO_4_, 2.0; (NH_4_)_2_SO_4_, 1.4; MgSO_4_ *·*7H_2_O, 0.3; CaCl_2,_ 0.3; yeast extract, 0.4; FeSO_4_ *·*7H_2_O, 0.005; MnSO_4_, 0.0016; ZnCl_2_, 0.0017; CoCl_2_, 0.002; CMC-Na, 5.0; and agar, 15.0; pH 5.0). After incubation at 28°C for 48 h, all the plates were stained with 1% (w/v) Congo-red solution for 15 min and discolored with 1 M NaCl for 15 min [19]. The degradation zones were visible around the bacteria, showing that the strains could hydrolyze CMC.

The modified Mandels medium (also called basal medium) used for CMCase production by the isolates contained the following components (in g/L: KH_2_PO_4_, 1.5; Na_2_HPO_4_ *·*7H_2_O, 2.5; (NH_4_)_2_SO_4_, 1.5; MgSO_4_ *·*7H_2_O, 0.3; CaCl_2_, 0.1; FeSO_4_ *·*7H_2_O, 0.005; MnSO_4_, 0.0016; ZnCl_2_, 0.0017; and CoCl_2_, 0.002; pH 7.0). The bacterial isolates were precultured overnight in general bacteria medium (in g/L: beef extract, 2; yeast extract, 2; sucrose, 6; and peptone, 5) at 28°C and 180 rpm. Subsequently, 2 mL of the culture was inoculated into 250 mL conical flask containing 50 mL of basal medium with 10 g/L of CMC-Na as the sole carbon source and incubated at 28°C and 180 rpm for 60 h.

### 2.3. Enzyme Assay

Enzyme production during cultivation was assayed at 12 h intervals up to 3 days. The cultures were centrifuged at 12,000 rpm for 10 min at 4°C. The supernatants were collected as crude enzyme for enzyme assay. CMCase, Avicel cellulase (Avicelase), and filter-paper cellulase (FPase) activities were determined using the 3,5-dinitrosalicylic acid (DNS) method [20]. The reaction systems were prepared as follows: 250 *μ*L of crude enzyme (appropriately diluted) mixed with 250 *μ*L of 2% (w/v) CMC for determining the CMCase activity; 500 *μ*L of enzyme mixed with 1 mL of Avicel (1%, w/v) for determining the Avicelase activity; and 500 *μ*L of enzyme mixed with 50 mg of Whatman number 1 filter paper (1.0 × 6.0 cm) in 1 mL of buffer for determining the FPase activity. The buffer used for dissolving or resuspending the substrates was 100 mM sodium citrate buffer (pH 5.5). The mixtures were incubated at 50°C for 30 min for CMCase assay and for 1 h for Avicelase and FPase assay, respectively. Then, the reactions were stopped by adding 1 mL of DNS reagent for CMCase assay and 3 mL of DNS reagent for Avicelase and FPase assay, respectively. All the mixtures were heated in boiling water for 5 min for color development. Subsequently, 200 *μ*L of each sample was transferred to 96-well microplate and the absorbance was measured at 540 nm [21, 22]. One unit (U) of the enzyme activity was defined as the amount of enzyme that released 1 *μ*mol of reducing sugars equivalent to glucose per minute during the reaction.

The activity of *β*-glucosidase was measured by using* p*-nitrophenyl-*β*-D-glucopyranoside (p-NPG) as substrate. The enzyme activity was determined by detecting the amount of* p*-nitrophenol (p-NP) produced from p-NPG [23]. One unit (U) of *β*-glucosidase activity was defined as the amount of enzyme liberating 1 *μ*mol of p-NP per minute.

### 2.4. 16*S* rRNA Gene Sequencing and Phylogenetic Analysis of the CMC-Degrading Isolates

The CMC-degrading isolates were cultivated in general bacteria medium at 28°C for 24 h. The culture was directly used for the amplification of bacterial 16S rRNA gene by PCR [24]. Two universal 16S rRNA gene primers (F27: 5′-AGAGTTTGATCCTGGCTCAG-3′ and R1492: 5′-TACGGTTACCTTGTTACGACTT-3′) were used [25]. The 25 *μ*L mixtures were composed of 1 *μ*L of bacterial culture as template DNA, 12.5 *μ*L of 2 × Taq PCR Master Mix (containing 0.5 U Taq DNA polymerase/*μ*L, 500 *μ*M of each dNTP, 20 mM Tris-HCl (pH 8.3), 100 mM KCl, 3 mM MgCl_2_, and bromophenol blue, purchased from Tiangen Biotech, Beijing, China), 1 *μ*L of each primer (10 *μ*M), and 9.5 *μ*L of double-distilled H_2_O. The PCR procedure employed was as follows: primary denaturation for 5 min at 94°C; 30 cycles of denaturation at 94°C for 30 s; annealing at 55°C for 30 s, and extension at 72°C for 100 s; and an additional reaction for 10 min at 72°C. The PCR products were detected on 0.8% agarose gel to confirm its purity, quantity, and size. The PCR products were sent to Sangon Biotech (Shanghai) Co., Ltd., China, for sequencing.

The 16S rRNA gene sequences were compared with other 16S rRNA gene sequences available in GenBank by using the BLASTN program (http://blast.ncbi.nlm.nih.gov/Blast.cgi) and aligned with similar sequences by using CLUSTX program. The phylogenetic tree was constructed by applying the neighbor-joining method using MAGA4.1 program based on Kimura-2 parameters with 1000 replicates of bootstrap values [26].

### 2.5. Morphological, Physiological, and Biochemical Identification of the Bacterial Strain ME27-1

The morphological properties of the strain ME27-1, including shape, size, colony characteristics (color, shape, surface, elevation, and edge), and Gram staining were evaluated [27]. The physiological and biochemical characterization of the strain ME27-1 was carried out by using API 50CHB microtests (bioMérieux).

### 2.6. Optimization of Cultivation Conditions for CMCase Production by the Strain ME27-1

The effect of initial pH and temperature on CMCase production by the strain ME27-1 was determined by cultivating the strain in 50 mL of basal medium containing 10 g/L of CMC-Na at various pH (ranging from 5.0 to 10.0 with an interval of 0.5) and temperatures (26–34°C) for 60 h at 180 rpm.

The effect of carbon and nitrogen sources on cellulase production by the strain ME27-1 was determined by using 11 different carbon sources (fructose, glucose, glycerol, lactose, sucrose, maltose, CMC-Na, filter paper (chopped into 20 mesh size), Avicel, soluble starch, and wheat bran which was chopped into 80 mesh size) and 10 different nitrogen sources as below: (NH_4_)_2_SO_4_, NH_4_NO_3_, NaNO_3_, KNO_3_, NH_4_Cl, urea, soybean, yeast extract, tryptone, and beef extract. The carbon sources were used at a concentration of 10 g/L, instead of the carbon source in the basal medium. Furthermore, different concentrations (10–100 g/L with an interval of 10 g/L) of optimal carbon source were examined. Similarly, the effect of nitrogen sources was also studied with an initial concentration of 1.5 g/L.

The effect of different inoculum sizes (2%, 4%, 6%, 8%, and 10%) on enzyme production was tested. All media were in pH 8.0. All the flasks were incubated at 28°C. The CMCase activity was detected at an interval of 12 h.

### 2.7. Properties of CMCase Produced by the Bacterial Strain ME27-1

To determine the optimal pH, 250 *μ*L of crude CMCase supernatant was incubated with 250 *μ*L of CMC-Na (2%, w/v) at 50°C and different pH (3.0–11.0 with an interval of 0.5), respectively. To observe the effect of temperature, CMCase was incubated with 2% CMC-Na at a pH of 5.5 and temperature ranging from 30 to 75°C with an interval of 5°C. The maximum CMCase activity obtained at different pH and temperatures was considered to be 100%.

The effect of pH on the stability of CMCase was studied by mixing the crude enzyme with different buffers (1 : 9, v/v) with pH ranging from 3.0 to 10.0. The CMCase activity of the crude enzyme after incubating at 4°C for 24 h at different pH was detected. To study the thermostability of the CMCase produced by the strain ME27-1, the crude enzyme was preincubated at different temperatures (varying from 30 to 75°C with an interval of 5°C) for 1 h. The residual CMCase activity was detected. The maximum CMCase activity obtained at pH 3.0–10.0 or temperature 30–75°C was considered to be 100%. All the enzyme assays were carried out in triplicate.

### 2.8. Nucleotide Sequence Accession Numbers

All the DNA sequences of the partial 16S rRNA genes of the 22 strains reported in this study have been deposited into the GenBank database under the accession numbers from KF536877 to KF536898.

## 3. Results and Discussion

### 3.1. Isolation and Screening of Cellulose-Degrading Bacteria

**Figure 1:**
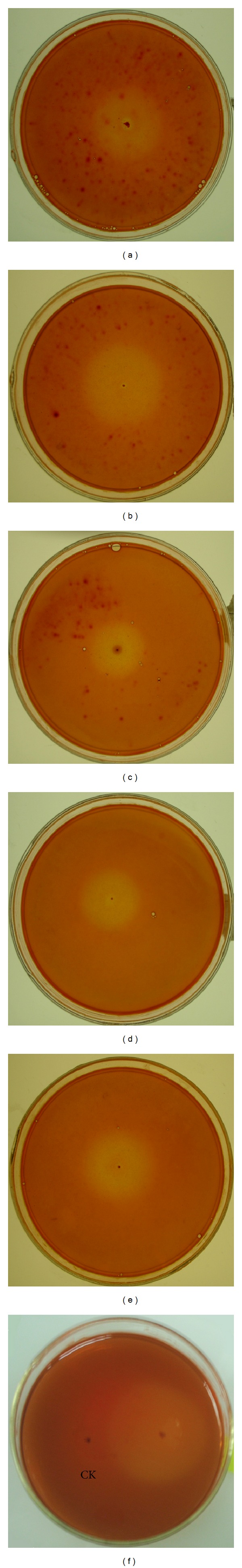
Hydrolyzing zones produced by bacterial strains on agar plates containing CMC after Congo-red staining. (a) Strain BS16-3, (b) strain FCD1-3, (c) strain FCD2-1, (d) strain FCD3-5, (e) strain FCD7-2, (f) strain SK3-4, and (CK)* Escherichia coli* DH5*α*.

A total of 245 cellulose-degrading aerobic bacterial strains were isolated from different natural reserves in the subtropical region of China, which were cultured in agar medium containing sugarcane bagasse pulp as the sole carbon source. Out of these strains, 22 isolates showed hydrolyzing zones on agar plates containing CMC-Na after Congo-red staining (Figure 1). The hydrolyzing zone diameter and colony diameter are listed in Table 1.

Among the 22 isolates, only three isolates (ME27-1, FCD1-3, and SK3-4) were found to produce measurable CMCase after liquid cultivation, and isolate ME27-1 showed the highest CMCase activity (0.17 U/mL) after incubation for 60 h in basal liquid medium containing 10 g/L of CMC-Na (Table 1). The CMCase activity of the other 19 strains was undetectable after cultivating in various liquid media for up to 6 days, and the Avicelase, FPase, and *β*-glucosidase activities of all the 22 bacterial strains were also undetectable.

Congo-red staining has been widely used in many studies for screening cellulose-degrading microorganisms. Although Teather and Wood described the relationship between the diameter of hydrolyzing zone and log enzyme concentration, this correlation could not represent the enzyme-producing ability of the microorganisms [19]. In the present study, although some strains presented large and clear hydrolyzing zones, the activities of CMCase and other cellulases produced by them were undetectable in various liquid media containing CMC and other cellulosic materials, suggesting that either the concentration of the enzyme produced by these strains was very low to be detected after cultivation in liquid medium or the ability of the strains to secrete CMCase was weak. Sadhu and Maiti also reported that the diameter of the hydrolyzing zone may not accurately reflect the real cellulase activity [28].

In general, aerobic bacteria produce low levels of Avicelase, FPase, and *β*-glucosidase. In a study carried out by Rastogi et al.,* Brevibacillus* sp. DUSELG12 and* Geobacillus* sp. DUSELR7 were found to produce a maximum FPase activity of 0.027 and 0.043 U/mL on days 7 and 8, respectively [12]. Recently, Soares et al. found that only 9.1% of bacterial strains were able to degrade Avicel on agar plates [7].

### 3.2. Identification of Cellulose-Degrading Bacteria

The DNA fragments containing partial 16S rRNA genes of the 22 isolates were amplified and sequenced. The sequences obtained were matched with those available in GenBank, which revealed maximum identity of these isolates and allowed identification of these cellulose-degrading bacterial strains (Table 1).

**Figure 2:**
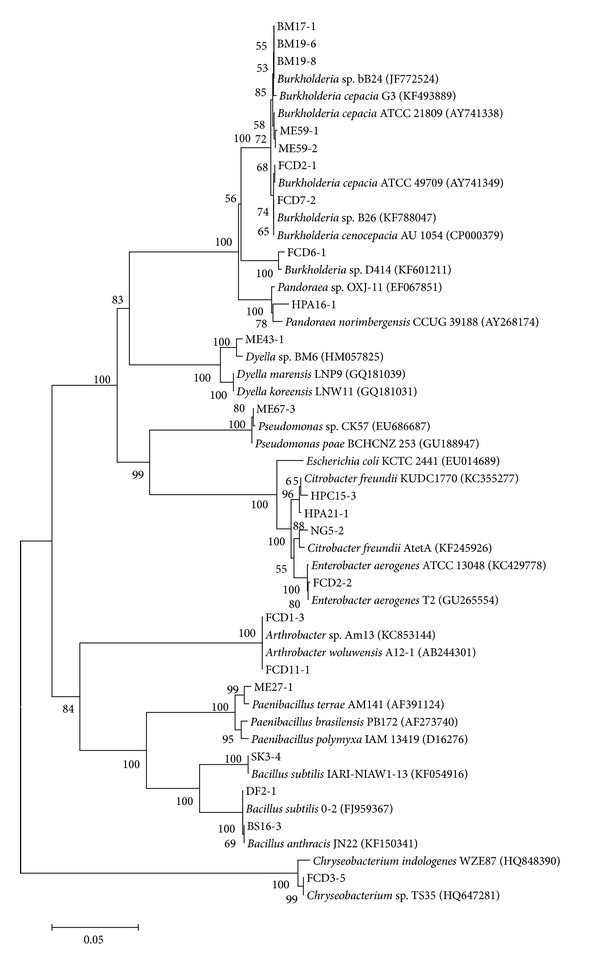
Phylogenetic tree for the 22 strains and related bacterial strains. The accession numbers of the strains are given in* brackets*.

It was found that the 22 aerobic bacterial strains that could hydrolyze cellulose belonged to 10 different genera:* Burkholderia* (36.36%),* Bacillus* (13.65%),* Citrobacter* (13.65%),* Arthrobacter* (9.10%),* Enterobacter* (4.54%),* Chryseobacterium* (4.54%),* Pandoraea* (4.54%),* Paenibacillus* (4.54%),* Dyella* (4.54%), and* Pseudomonas* (4.54%). The phylogenetic tree of the 22 strains was constructed by using MAGA4.1 program (Figure 2).

Various cellulose-degrading bacteria have been found in different environments. The genus* Burkholderia* was observed to be the main cellulose-hydrolyzing bacteria and was considered to play an important role in cellulose degradation in the subtropical region of China in this study. In addition, bacteria belonging to the genera* Arthrobacter*,* Chryseobacterium*,* Pandoraea*, and* Dyella* were also found to be cellulolytic in the present study, which have been rarely reported as cellulose-degrading bacteria. In a previous study, Lo et al. reported that the cellulase-producing bacterial strains isolated from a rice field in southern Taiwan mainly belonged to the genus* Cellulomonas* [9]. On the other hand,* Bacillus* was reported to be the dominant cellulose-degrading bacteria in samples collected from paper mill sludges and organic fertilizers from Red Rock, Canada, as well as in those from soil, compost, and animal waste slurry from Jeju Island [29, 30]. Similarly,* Burkholderia* was found to be the main genus of cellulase-producing bacteria in the subtropical rainforest in Okinawa Island, Japan [31].

The strain ME27-1, with higher CMCase activity, was thoroughly examined. The partial 16S rRNA gene (1309 bp) from the strain ME27-1 showed a maximum identity of 99% with that of* Paenibacillus terrae* AM141^T^ (T: type strain). Morphological tests revealed that the cells of the strain ME27-1 were rod-shaped, endospore-forming, Gram-positive, and 0.8 × 1.9–3.2 *μ*m in size. The appearance of the colony on the TSA medium was cream-colored, moist, irregular, swollen, and pigment-free. The biochemical properties of the strain ME27-1 are listed in Table 2. The morphological, physiological, and biochemical properties of the strain ME27-1 were found to be mostly similar to those of* P. terrae* [27]. Thus, the strain ME27-1 was identified as* P. terrae*.

To our knowledge, till date, no study has reported about CMCase production by* P. terrae*, although other species of* Paenibacillus* have been found to produce cellulase. Some CMCase genes cloned from* Paenibacillus polymyxa* GS01,* Paenibacillus barcinonensis*,* Paenibacillus xylanilyticus* KJ-03, and* Paenibacillus cookii* SS-24 have been expressed in* Escherichia coli* and* Saccharomyces cerevisiae* [32–35]. On the other hand, CMCases from* Paenibacillus curdlanolyticus* B-6,* Paenibacillus campinasensis* BL11,* Paenibacillus* sp. B39, and* P. polymyxa* have been purified [36–39].

### 3.3. Effect of Initial pH, Temperature, Carbon and Nitrogen Sources, Inoculum Size, and Incubation Time on CMCase Production by* P. terrae* ME27-1

**Figure 3:**
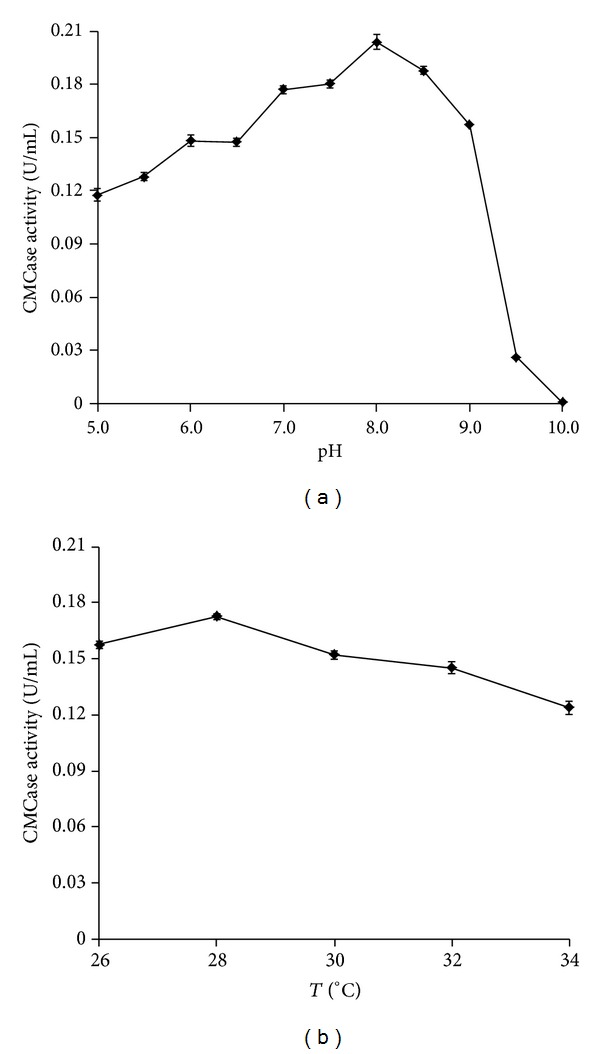
Effect of initial pH and temperature on enzyme production by the strain ME27-1.** (**a) Initial pH. (b) Temperature (*T*).

The best incubation conditions were pH 8.0 and 28°C (Figures 3(a) and 3(b)). The CMCase activity declined when the initial pH and incubation temperature were not optimal. There have been diverse reports on the optimal initial pH and temperature for cellulolytic enzyme production by* Paenibacillus* sp. In a previous study,* P. curdlanolyticus* B-6 was cultivated for enzyme production at pH 7.0 and 37°C [5]. Furthermore, Kumar et al. reported that the optimal initial pH and temperature for CMCase production by* P. polymyxa* were 5.5 and 37°C, respectively [39]. Yoon et al. accounted that the optimal growth temperature for* P. terrae* was 30°C, which is similar to that observed for optimal CMCase production by the strain ME27-1 [27].

**Figure 4:**
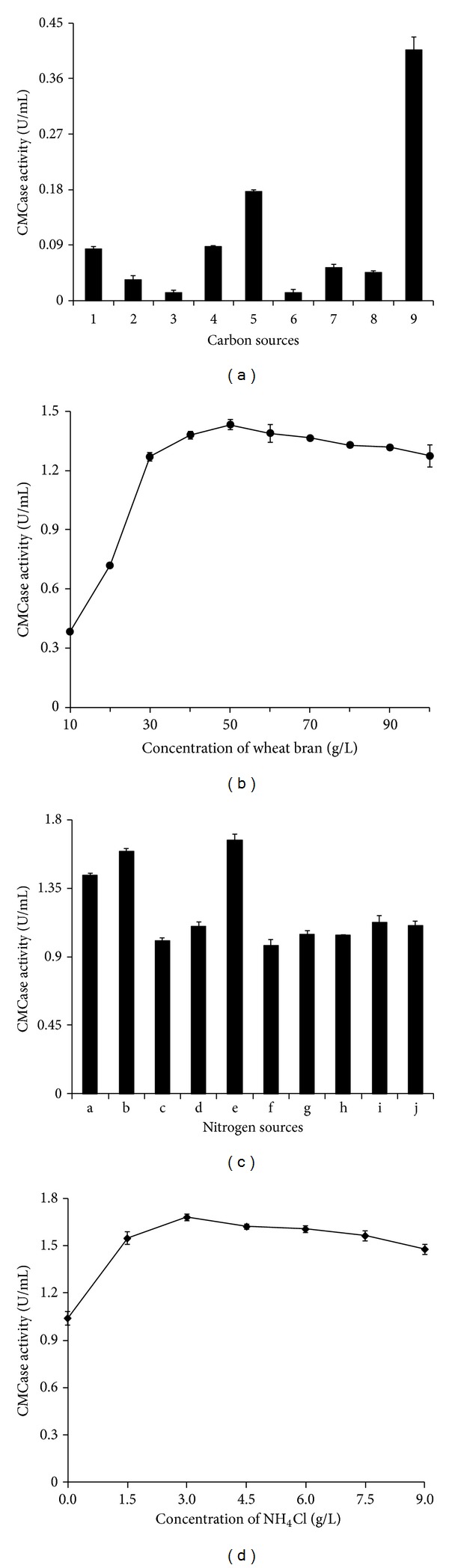
Effect of carbon and nitrogen sources on CMCase production by the strain ME27-1. (a) Different carbon sources: 1 ~ 9 represented glycerol, lactose, sucrose, maltose, CMC-Na, filter paper, Avicel, soluble starch, and wheat bran, respectively. (b) The concentration of wheat bran. (c) Different nitrogen sources: a ~ j represented (NH_4_)_2_SO_4_, NH_4_NO_3_, NaNO_3_, KNO_3_, NH_4_Cl, urea, soybean, yeast extract, tryptone, and beef extract, respectively. (d) The concentration of NH_4_Cl.

Various cellulosic materials have been used to induce microorganisms to produce cellulase. When fructose and glucose were used as the sole carbon source, no CMCase activity was detected. Wheat bran induced the highest CMCase activity, which was about 2.5-fold higher than that observed in the basal medium containing CMC-Na (Figure 4(a)). The optimal concentration of wheat bran in the medium was found to be 50 g/L (Figure 4(b)). Da Vinha et al. used steam-pretreated sugarcane bagasse (or wheat bran) as the main carbon source and found that wheat bran was the best inducer for CMCase production by* S. viridobrunneus* SCPE-09 [15]. Gao et al. demonstrated that rice bran was the optimal carbon source for CMCase production by* Cellulophaga lytica* LBH-14, while Kumar et al. reported that high CMCase production by* P. polymyxa* was obtained when using mango peel as substrate [39, 40]. In addition, wheat straw, rice straw, and xylan have been reported to be good carbon sources for CMCase production by* Cellulomonas* sp. and* Cellulosimicrobium cellulans* [9, 41].

Furthermore, maximum CMCase activity was noted when using NH_4_Cl as the sole nitrogen source (Figure 4(c)), and the best concentration of NH_4_Cl in the medium was observed to be 3 g/L (Figure 4(d)). Many reports have shown that organic nitrogen sources are better than inorganic nitrogen sources [15, 16, 42, 43]. In the present study, the CMCase activity of the strain ME27-1 was higher when inorganic nitrogen sources were used as the sole nitrogen source. Likewise, Kumar et al. and Kalogeris et al. also observed a similar phenomenon in their studies [39, 44].

**Figure 5:**
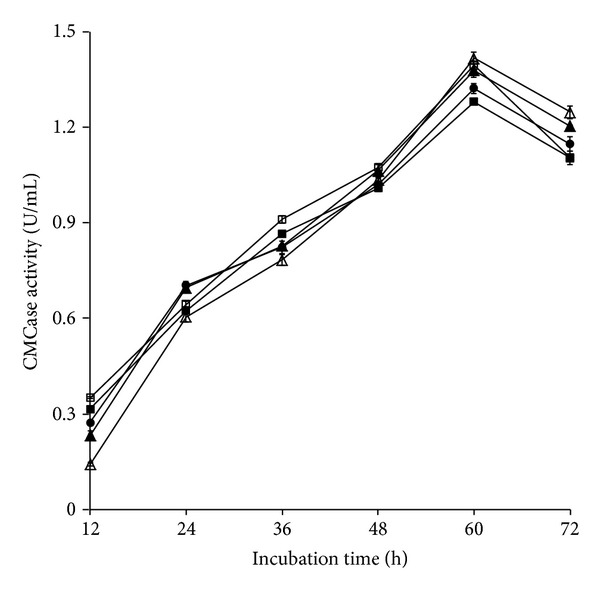
Effect of inoculum size and incubation period on CMCase production by the strain ME27-1. 2% (*empty triangle*); 4% (*filled triangle*); 6% (*filled circle*); 8% (*filled square*); and 10% (*empty square*).* Error bars* show the standard deviation of experimental point (*n* = 3).

In addition, use of an inoculum size of 2% resulted in maximum CMCase activity after incubation of the strain for 60 h (Figure 5). There has been increasing interest in cellulase-producing bacteria because of their ability to grow fast [45]. In the present study, the strain ME27-1 produced the highest CMCase activity after 60 h of incubation. On the other hand, in previous studies, maximum CMCase activity of* Pseudomonas* sp. HP207 and* S. viridobrunneus* SCPE-09 was observed after 24 and 48 h of incubation, respectively, which is much earlier than that noted for the strain ME27-1 [15, 16]. However, different results have been reported in various studies. Maximum CMCase activity of* C. lytica* LBH-14 was obtained after 72 h of incubation, whereas that of* Brevibacillus* sp. DUSELG12 and* Geobacillus* sp. DUSELR7 was noted after days 9 and 7, respectively [12, 40].

### 3.4. Properties of CMCase Produced by* P. terrae* ME27-1

**Figure 6:**
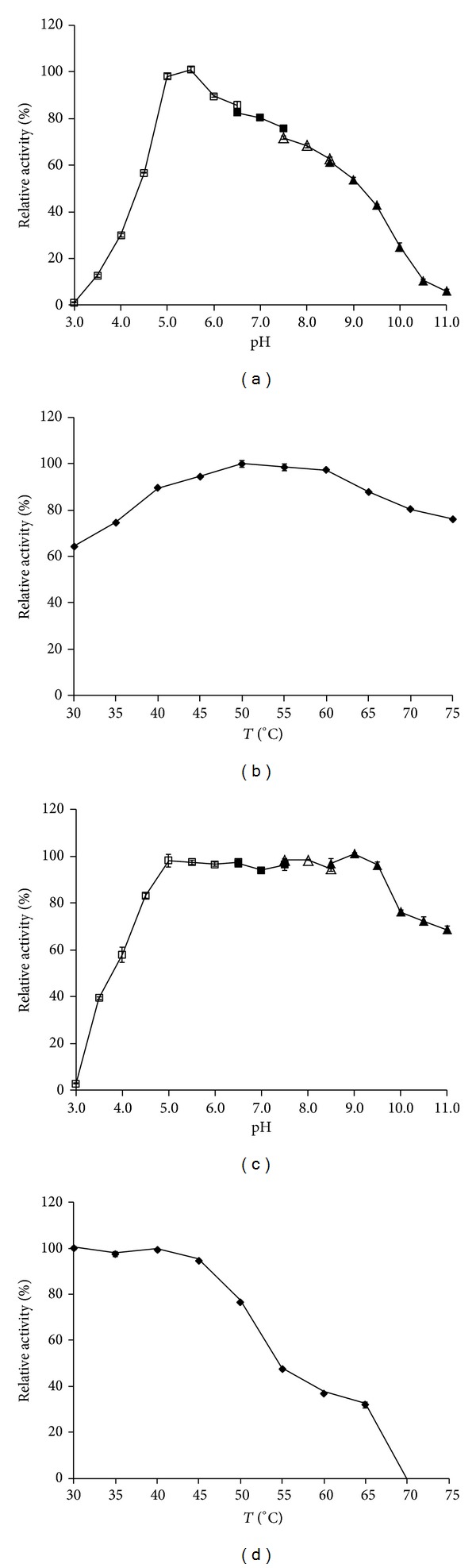
Properties of CMCase produced by the strain ME27-1. (a) Effect of pH on CMCase activity. (b) Effect of temperature on CMCase activity. (c) Effect of pH on the stability of CMCase. (d) Thermostability of CMCase. The different buffers used are as follows (100 mM): sodium citrate buffer (*empty square*; pH 3.0–6.5), Na_2_HPO_4_-NaH_2_PO_4_ buffer (*filled square*; pH 6.5–7.5), Tris-HCl buffer (*empty triangle*; pH 7.5–8.5), and glycine-NaOH buffer (*filled triangle*; pH 8.5–11.0).* Error bars* show the standard deviation of experimental point (*n* = 2).

The optimum pH and temperature of CMCase produced by strain ME27-1 were found to be 5.5 and 50°C, respectively (Figures 6(a) and 6(b)). The CMCase produced by the strain ME27-1 was stable from pH 4.0 to 11.0, with more than 60% CMCase activity being retained (Figure 6(c)). Furthermore, the enzyme maintained 65% activity after incubation at 4°C and pH 11.0 for 24 h. The temperature profiles demonstrated that more than 95% CMCase activity was retained at 30–45°C for 1 h (Figure 6(d)). However, the enzyme activity was reduced at temperatures above 50°C. In fact, approximately 77% residual activity was maintained after preincubating the enzyme at 50°C for 1 h.

Similar results were observed for cellulases produced by* S. viridobrunneus* SCPE-09 and* P. cookii* SS-24, with an optimal pH of 5.0 and 5.1 and an optimal temperature of 50° and 55°C, respectively [15, 35]. However, maximum CMCase activity of bacteria at pH lower than 6.0 has been rarely observed, and the maximum CMCase activities of* P. campinasensis* BL11,* P. polymyxa* GS01,* Paenibacillus* sp. B39, and* Bacillus mycoides* S122C were observed at neutral or alkaline conditions [37, 38, 46, 47]. In the present study, the CMCase produced by the strain ME27-1 was stable at pH 5.0–9.5, and almost 85% residual activity was retained. Only a few studies have reported that CMCase was stable at such a wide pH range; for example, Da Vinha et al. reported the 60% CMCase activity was retained within the pH range of 3.0–8.0 [15].

### 3.5. Comparison of CMCase Production by* P. terrae* ME27-1 and Other Microorganisms

When measured at the optimal pH and temperature of CMCase,* P. terrae* ME27-1 produced CMCase activity of 2.08 U/mL under the optimized cultivation conditions, which was a 12-fold improvement in the CMCase production. This yield of CMCase production was higher than most of the aerobic bacterial strains but less than some of aerobic bacterial strains that have been exploited previously (Table 3). However, the CMCase production by* P. terrae* ME27-1 was lower than that by several anaerobic bacterial strains, for example,* Clostridium papyrosolvens* CFR-703,* C. thermocellum* YM4,* C. thermocopriae* JT3-3 (Table 3). Some anaerobic bacteria can degrade lignocellulosic substrates efficiently by producing multienzyme complex termed cellulosome [36]. The carbohydrate binding modules and different proteins in the cellulosome allow the whole enzyme complex to bind to the substrates, which avoids the wasteful expenditure of energy of bacteria releasing large amounts of individual enzymes and makes lots of advantages over single-enzyme system [4, 48].

Furthermore, the CMCase produced by* P. terrae* ME27-1 was lower than that by most aerobic fungal strains while it was higher than that by anaerobic fungal strains (Table 3). The CMCase production by most bacteria was usually lower than that by aerobic fungal strains. Genomic analysis showed that less glycosyl hydrolases existed in aerobic bacterial strains than aerobic fungal strains, which may explain why aerobic bacteria usually produce lower CMCase activity [48].

## 4. Conclusion

Ten genera of bacteria hydrolyzing cellulose were isolated from different natural reserves in the subtropical region of China, and the genus* Burkholderia* was found to be the most prevalent and predominant. The strain ME27-1, identified to be* P. terrae*, showed the highest CMCase activity among the 22 strains isolated, and after optimization of the cultivation conditions, the enzyme activity was significantly improved to 2.08 U/mL. This bacterial species has been rarely found to produce cellulase. Thus, this study revealed the diversity of cellulose-degrading bacteria in the subtropical region of China and found that* P. terrae* ME27-1 was a good CMCase producer.

## Figures and Tables

**Table 1:** Cellulose-degrading bacteria isolated from different natural reserves of subtropical region in China.

Strains	Location	*D*/*d* (mm)	CMCase activity (U/mL)	Max identity (%)	Strain of closest match	Identification
BM17-1	Baima Snow Mountains	24/1.9	ND	99	*Burkholderia* sp. bB24(JF772524)	*Burkholderia *
BM19-6		23/1.8	ND	99	*Burkholderia* sp. bB24(JF772524)	*Burkholderia *
BM19-8		25/2.3	ND	99	*Burkholderia* sp. bB24(JF772524)	*Burkholderia *

BS16-3	Dawang Ridge	30/5	ND	99	*Bacillus anthracis *JN22(KF150341)	*Bacillus *

DF2-1	Nanning city	31/2.7	ND	99	*Bacillus subtilis *0–2 (FJ959367)	*Bacillus *

FCD1-3	Fangchenggang city	34/2	0.06 ± 0.002	99	*Arthrobacter* sp. Am13(KC853144)	*Arthrobacter *
FCD2-1		20/2.2	ND	100	*Burkholderia cepacia *ATCC 49709(AY741349)	*Burkholderia *
FCD2-2		25/2.4	ND	99	*Enterobacter aerogenes *T2(GU265554)	*Enterobacter *
FCD3-5		28/3	ND	99	*Chryseobacterium *sp. TS35(HQ647281)	*Chryseobacterium *
FCD6-1		20/2	ND	99	*Burkholderia *sp. D414(KF601211)	*Burkholderia *
FCD7-2		28/2.6	ND	99	*Burkholderia *sp. B26(KF788047)	*Burkholderia *
FCD11-1		24/1.5	ND	99	*Arthrobacter woluwensis *A12-1(AB244301)	*Arthrobacter *

HPA16-1	Huaping	24/2.5	ND	98	*Pandoraea norimbergensis *CCUG 39188(AY268174)	*Pandoraea *
HPA21-1		30/2.3	ND	99	*Citrobacter freundii *KUDC1770(KC355277)	*Citrobacter *
HPC15-3		25/2	ND	98	*Citrobacter freundii *KUDC1770(KC355277)	*Citrobacter *

ME27-1	Maoer Mountains	30/3	0.17 ± 0.005	99	*Paenibacillus terrae* AM141(AF391124)	*Paenibacillus terrae *
ME43-1		29/3.5	ND	99	*Dyella *sp. BM6(HM057825)	*Dyella *
ME59-1		29/2.7	ND	99	*Burkholderia cepacia *ATCC 21809(AY741338)	*Burkholderia *
ME59-2		26/2.5	ND	99	*Burkholderia cepacia *ATCC 21809(AY741338)	*Burkholderia *
ME67-3		31/3.4	ND	99	*Pseudomonas *sp. CK57(EU686687)	*Pseudomonas *

NG5-2	Longgang	20/2	ND	99	*Citrobacter freundii *AtetA(KF245926)	*Citrobacter *

SK3-4	Shankou Halodrymium	43/4.6	0.01 ± 0.001	99	*Bacillus subtilis* IARI-NIAW1-13(KF054916)	*Bacillus *

“*D*/*d*”: hydrolyzed zone diameter/colony diameter on agar media containing CMC as sole carbon source; “ND”: no detectable activity.

**Table 2:** Physiological and biochemical properties of strain ME27-1.

Characteristics	Reaction
Motility	+
Catalase	+
H_2_S production	−
Nitrate reduction	+
Hydrolyzing ability	
Starch	+
Gelatin	+
Acid fermentation	
Glycerol	−
Ribose	+
*β*-Methyl-D-xyloside	−
Mannose	+
Inositol	−
*α*-Methyl-glucoside	+
Esculin	+
Lactose	+
Synanthrin	−
Glycogen	+
D-Lyxose	−
D(L)-Arabitol	−
5-Keto-gluconate	−
Erythritol	−
D-Xylose	+
Galactose	−
Sorbose	−
Mannitol	−
N-Acetyl-glucosamine	−
Salicine	+
Melibiose	+
Melezitose	−
Xylitol	−
D-Tagatose	−
D-Arabinose	−
L-Xylose	+
Glucose	−
Rhamnose	−
Sorbitol	−
Amygdalin	+
Cellobiose	+
Sucrose	+
Raffinose	+
Gentiobiose	+
D-Fucose	−
Gluconate	−
L-Arabinose	+
Adonitol	−
Fructose	+
Dulcitol	−
*α*-Methyl-D-xyloside	−
Arbutin	+
Maltose	+
Trehalose	+
Starch	+
D-Turanose	−
L-Fucose	−
2-Keto-gluconate	−

“+”: positive reaction; “−”: negative reaction.

**Table 3:** Comparison of CMCase production by *Paenibacillus terrae* ME27-1 with other bacterial and fungal strains.

Strains	Carbon source	Nitrogen source	Aerobic/anaerobic	CMCase activity (U/mL)	Ref.
***P. terrae* ME27-1**	**Wheat bran**	**N** **H**_4_ **C** **l**	**Aerobic**	**2.08**	**This study**
*Acinetobacter anitratus *	CMC	(NH_4_)_2_SO_4_	Aerobic	0.48	[11]
*Branhamella* sp.	CMC	(NH_4_)_2_SO_4_	Aerobic	2.56	[11]
*Bacillus subtilis* AS3	CMC	Peptone, yeast extract	Aerobic	0.43	[14]
*B. pumilus* EWBCM1	Galactose	Malt extract, H_8_MoN_2_O_4_	Aerobic	0.58	[49]
*B. pumilus* BpCRI 6	CMC, glycerol	Tryptone	Aerobic	1.90	[50]
*Pseudomonas* sp. HP207	CMC–Na	Yeast extract	Aerobic	1.43	[16]
*Streptomyces viridobrunneus *SCPE-09	Wheat bran	Corn steep liquid	Aerobic	2.00	[15]
*S. drozdowiczii *	CMC	Yeast extract	Aerobic	0.59	[51]
*Streptomyces* sp. J2	Starch, glucose	NH_4_Cl	Aerobic	0.43	[52]
*Streptomyces* sp. SLBA-08	Sisal bagasse	(NH_4_)_2_SO_4_	Aerobic	1.11	[53]
*S. griseoaurantiacus * ZQBC691	CMC	(NH_4_)_2_SO_4_	Aerobic	37.38	[54]
*Clostridium thermocellum * YM4	Solka floe	NH_4_Cl	Anaerobic	6.70	[55]
*C. thermocopriae* JT3-3	Cellulose MN300	Yeast extract, urea	Anaerobic	4.53	[56]
*C. papyrosolvens* CFR-703	Cellulose	Yeast extract	Anaerobic	45.00	[57]
*Geobacillus* sp. T1	Barley straw	NH_4_Cl	Aerobic	143.50	[58]
*Chaetomium globosum* 414	OPEFB	Peptone	Aerobic	30.80	[59]
*Chalara paradoxa* CH32	Glucose	Malt extract, yeast extract	Aerobic	0.25	[60]
*Aspergillus awamori *2B.361 U2/1	Wheat bran	Yeast extract, NaNO_3_	Aerobic	4.90	[61]
*Trichoderma reesei* RUT-C30	Wheat bran	Yeast extract, NaNO_3_	Aerobic	20.00	[61]
*Penicillium janthinellum* NCIM 1171	CP-123	(NH_4_)_2_SO_4_	Aerobic	111.80	[62]
*T. viride* NCIM 1051	CP-123	(NH_4_)_2_SO_4_	Aerobic	140.70	[62]
*P. decumbens* JU-A10	Wheat bran	NaNO_3_, urea	Aerobic	10.60	[63]
*P. pinophilum *	Wheat bran	(NH_4_)_2_SO_4_	Aerobic	65.00	[64]
*Neocallimastix *sp. R1	Wheat straw	Trypticase peptone, NH_4_Cl	Anaerobic	0.19	[65]
*N. frontalis* PN-1	Filter paper strip	(NH_4_)_2_SO_4_	Anaerobic	0.94	[66]
*Neurospora crassa *	Wheat straw	Yeast extract	Aerobic	19.70	[67]
*Trichoderma* sp. A-001	Filter paper	KNO_3_	Aerobic	167.00	[68]
*Volvariella volvacea *	Avicel	Yeast extract, NH_4_NO_3_	Aerobic	0.64	[69]

CMC: carboxymethyl cellulose; OPEFB: oil palm empty-fruit-bunch fibres; CP-123: cellulose powder 123.
